# Concentrations of escitalopram in blood of patients treated in a naturalistic setting: focus on patients with alcohol and benzodiazepine use disorder

**DOI:** 10.1007/s00406-022-01491-9

**Published:** 2022-10-07

**Authors:** X. M. Hart, S. Heesen, C. N. Schmitz, S. Dörfler, D. Wedekind, G. Gründer, C. Hiemke, U. Havemann-Reinecke

**Affiliations:** 1 Department of Molecular Neuroimaging, Medical Faculty Mannheim, Central Institute of Mental Health, Heidelberg University, J5, 68159 Mannheim, Germany; 2 Department of Psychiatry and Psychotherapy, University Medical Center Göttingen, University of Göttingen, Göttingen, Germany; 3 Institute of Clinical Chemistry and Laboratory Medicine, Department of Psychiatry and Psychotherapy, University Medical Center of Mainz, Mainz, Germany

**Keywords:** SSRI, Escitalopram, Depressive disorder, Depression, Pharmacokinetics, Alcohol use disorder, Benzodiazepine use disorder

## Abstract

The selective serotonin reuptake inhibitor escitalopram (ESC) is indicated for the treatment of major depressive disorder (MDD) and of generalized anxiety disorder (GAD). Monitoring of blood levels (BLs) is strongly indicated due to ESC’s high interindividual pharmacokinetic variability. The aim of this study was to analyse clinical efficacy and pharmacokinetic influences on ESC BLs, in patients with depressive disorder alone and with comorbid alcohol or benzodiazepine use disorder. Data were collected from patients treated under naturalistic conditions for whom Therapeutic Drug Monitoring (TDM) was requested to guide antidepressant drug therapy and analysed retrospectively. Particular emphasis was given to patients with alcohol or benzodiazepine use disorder. Responders according to the clinical global impression (CGI) scale were compared with nonresponders for their ESC blood level (BL). The patient sample included 344 patients from 16 psychiatric hospitals in Germany. Influencing factors that could explain 22% of ESC BLs were dose, sex and age. Variability was high between individuals, and doses up to 40 mg were common in real-world settings. Patients treated with ESC monotherapy who responded showed a trend towards higher BLs compared to nonresponders with a concentration of 15 ng/mL separating both groups. Pathological changes in liver function (indicated by elevated GGT in combination with an AST/ALT ratio ≥ 1) resulted in higher dose-corrected ESC concentrations. Influencing factors that could explain 22% of ESC blood levels were dose, sex, and age. Our findings confirm the currently recommended lower threshold level and support the need for standard TDM analyses in everyday clinical practice. The ICD 10 diagnosis alcohol dependence alone does not lead to pharmacokinetic changes in the metabolism of ESC, but altered liver function does.

**Supplementary Information:**

The online version contains supplementary material available at 10.1007/s00406-022-01491-9.

## Background

Prescription rates of citalopram’s racemic S-isomer escitalopram (ESC) has been forged ahead in the past years [1]. ESC has become a popular alternative to its precursor citalopram owed to ESC’s convincingly proven antidepressant effect and tolerability profile. The selective serotonin reuptake inhibitor (SSRI) is indicated for the treatment of major depressive disorder (MDD) and of generalized anxiety disorder (GAD). It is also approved for the treatment of obsessive compulsive disorder (OCD) in the EU, but not in the USA. The approved ESC doses range from 10 to 20 mg per day. Under naturalistic conditions, however, up to 40 mg/day are common accounting for a high interindividual pharmacokinetic variability [2–5]. Despite the manufacturer’s notifications about the influence of population-dependent influences such as older age and hepatic dysfunction on ESC pharmacokinetics [6], data from naturalistic patient populations is surprisingly rare. Among previously published studies, two important factors on ESC drug concentrations, age [2–4, 7] and sex [3, 7–9], have been frequently discussed. However, findings are inconsistent [2, 5]. ESC is primarily metabolized by the cytochrome P450 (CYP) isoenzymes CYP2C19 (36%), CYP2D6 (30%) and CYP3A4 (34%). Two major metabolites, S-desmethylescitalopram (S-DCT) and S-didesmethylescitalopram (S-DDCT), have been identified, which weakly contribute to the pharmacologic activity of ESC. On this account, CYP2C19 genotypes have been shown to substantially impact ESC levels [4, 7, 8, 10, 11]. Little information is known about the influence of prescribed comedication [2]. No information is available on the influence of liver abnormalities e.g. caused by alcohol abuse, a common comorbidity in patients treated with ESC for MDD, GAD or OCD. Furthermore, very few studies systematically investigated antidepressant effects or side effects of ESC in relation to drug levels [12, 13]. In a naturalistic setting, only one TDM study reported drug effects from a small sample of ten ESC treated patients [14]. Overall, limited data are available describing the relationship between ESC BLs, medication efficacy and tolerability. Nevertheless, current guidelines recommend BL monitoring for ESC for dose titration, special indications and for problem solving, and they suggest a reference range between 15 and 80 ng/mL [15]. This is the first study that investigates drug levels and clinical efficacy in a large sample of patients treated with ESC in a naturalistic setting. The aim of our study was to investigate an optimal concentration range for ESC and identify influences on ESC BLs.

## Material and methods

### Patient sample

Influencing factors like age, sex, comedication, liver function (GGT and AST/ALT ratio), comorbid alcohol-related disorder (International Classification of Diseases (ICD) 10 F10.2; F10.3) and benzodiazepine-related disorder (ICD 10 F 13.2.; 13.3) affecting the pharmacokinetics of ESC were studied in a naturalistic design. Data were collected between 08 January 2004 to 07 September 2009 from patients for whom TDM was requested to guide the antidepressant drug therapy in sixteen Departments of Psychiatry and Psychotherapy in Germany (Aachen, Augsburg, Bad Soden, Dresden, Göttingen, Gummersbach, Heidelberg, Karlsbach-Langensteinbach, Kiedrich, Königstein im Taunus, Mainz, Marienheide, München, Nürnberg, Ulm, Wasserburg). Alcohol- and benzodiazepine-dependent patients were inpatients for a qualified withdrawal treatment for at least three weeks. They were treated for a psychiatric disorder, for which treatment with ECS was indicated. Drug levels, demographic data, daily dose, diagnoses (according to the 10^th^ edition of the International Classification of Diseases (ICD-10) [16]), comedication, laboratory results, the reason for therapeutic drug monitoring (TDM), the severity of illness, therapeutic effects and side effects were registered on the request form by the requesting physician. Side effects were rated using a short version of the Utvalg for Kliniske Undersogelser (UKU [17]) rating scale with a four-point global scale (0, absent; 1, mild; 2, moderate; 3, severe) for severity on the day of blood withdrawal. Severity of illness and the patient’s response were assessed on the day of blood withdrawal with the Clinical Global Impressions Scale (CGI-I [18]), item 1 for evaluation of severity of illness (from score 2–8) and item 2 as global improvement rating (1, very much improved; 2, much improved; 3, slightly improved; 4, unchanged or worse). Minimal drug concentrations (trough levels) of ESC and two metabolites were measured under steady-state conditions from patients whose treatment was guided by TDM. Patients with doses ranging from 5–40 mg per day were eligible for analysis. Only one level per patient was selected, the last sample for which the daily dose was given on the request form. Reasons for exclusion of individual data were: i) missing information on administered ESC dose, ii) no escitalopram was detectable (0 ng/ml), iii) citalopram noted as comedication, iv) drug concentration was not in the steady-state, v) sample not taken at trough vi) chromatographic interferences, vii) noncompliance was reported by the clinician on the request form and viii) questionable compliance documented by the clinician and patients below individual dose-related reference range for both ESC and D-ESC.

### Determination of blood levels

ESC and two major metabolites D-ESC and DD-ESC were determined in serum by high performance liquid chromatography (HPLC) as described previously for mirtazapine [19] with slight modifications in the Neurochemical Laboratory of the Department of Psychiatry and Psychotherapy University Medical Center at Mainz, Germany. An HPLC system (Agilent 1100 obtained from Bio-Rad, Munich, Germany) with column-switching was used consisting of an autosampler, a thermostated column set at 25 °C with an electric six-port switching valve, two HPLC pumps and a fluorescence detector. For online sample clean-up, 0.1 ml serum was injected on a pre-column (10 × 4.0 mm i.d.) filled with LiChrospher CN material of 20 µm particle size (MZ-Analysentechnik, Mainz, Germany). The pre-column was washed with deionized water containing 8% (V/V) acetonitrile to remove proteins and other interfering compounds for five minutes. Drugs were eluted and separated on LiChrospher CN material (5 µm; column size 250 × 4.6 mm i.d., MZ-Analysentechnik) using 50% (V/V) acetonitrile and phosphate buffer (8 mM, pH 6.4) and quantified by fluorescence detection. The excitation wavelength was set at 290 nm, and the emission wavelength at 350 nm. HPLC analysis of a single sample was completed within 20 min. Each analytical series included at least two control samples containing a low or high concentration of ESC and D-ESC, respectively. There was linear relation between drug concentration and detector signal from 2 to at least 200 ng/mL. The lower limit of quantification was 2 ng/mL. The intra- and inter-assay reproducibility of quality control samples were below 10% for all analyses. For calculations, results reported as < 5 ng/mL and < 10 ng/mL (*n* = 16) were set to 2.5 and 5 ng/mL.

### Statistical analysis

#### Antidepressant effects and side effects

Escitalopram medication effects were investigated i) in a sample of patients with depressive disorder under CNS-relevant comedication and ii) in a sample of patients without CNS-relevant comedication. Responders were identified as patients with CGI-Improvement score ≤ 2. Nonresponders were characterized as patients with CGI-Improvement score > 2 or nonresponse noted as reason for TDM on the request form. A Kruskal–Wallis test was applied to compare drug levels among patient groups. Receiver operating characteristic (ROC) analysis was used to define a drug level threshold that is able to distinguish responders from nonresponders. Calculations were carried out using SPSS (version 26) and R 2.10.1. For all analyses, *p* ⩽ 0.05 was defined as statistically significant.

#### Identification of factors influencing ESC blood levels

Pharmacokinetic variability of ESC was expressed as the range in dose-adjusted serum concentrations (C/D ratios; ng/mL/mg/day). As an in vivo measure of CYP activities, the metabolic ratios D-ESC/ESC and DD-ESC/ESC were calculated. For descriptive analyses, mean, median, standard deviation and interquartile range were calculated. Differences between males and females and different age groups were tested by a two-tailed, nonparametric Mann–Whitney test, and for multiple comparisons, the Kruskal–Wallis test with Dunn’s post hoc test was performed. Correlation coefficients (Spearman-rho) were calculated to determine the relation between drug serum levels, daily doses, age and liver function (estimated by γ-glutamyltransferase (GGT), alanine aminotransferase (ALT) and ratio of aspartate aminotransferase (AST)/ALT). An AST/ALT ratio ≥ 1 with concurrently increased AST/ALT has been associated with the incidence of liver cirrhosis. Together with an elevated GGT, it has been found a quite selective parameter indicating an alcoholic liver disease [20]. However, contradicting evaluations of liver parameters were reported, which clearly indicate liver abnormalities that influence the drug serum levels. In this study, liver dysfunctions were assumed in patients that showed a GGT (66 U/l for men, 39 U/l for women) value above the recommended reference range and additionally an AST/ALT ratio ≥ 1 (with and without increased AST/ALT).The comparison group comprised patients with GGT values within the recommended reference range for women and men. In a similar manner, patients with an alcohol or benzodiazepine dependence were compared to a control group in order to determine a possible role of liver dysfunctions on the pharmacokinetics of ESC. We then used a multivariate modelling approach to predict ESC concentration based on clinical parameters. Pharmacokinetically relevant variables such as ESC dose, age, sex and comedication with cytochrome CYP2D6 inhibitors were used to predict the ESC concentration of each subject. For this analysis, we used generalized linear models (GLM) with a linear link-function and a gamma distribution underlying the response variable. Dose, age and sex were selected as predictors for the GLM since patients with CYP altering comedication were sparse. The modelling was performed using the custom written python-code as well as the sklearn-toolbox [21].

## Results

### Patient sample characteristics

Of 344 patients, 44 were excluded (39 patients without indication of doses, 2 outliers excluded, 1 patient with citalopram comedication and 2 patients with doses more than twice above the approved maximum daily dosage (> 40 mg)). The final sample comprised 300 patients that were included in the analysis (female: *n* = 180, 60%; male: *n* = 119, 39.7%, unknown *n* = 1) aged from 18 to 86 years (mean 48.6 ± 16.5 years). Most patients were from the University Medicine of Göttingen (36.3%), followed by Mainz (24.3%), Ulm (15.7%), and Augsburg (14.0%). For 178 patients, information on patient setting was available with most of patients staying in a psychiatric hospital at the time of inclusion (89.9%). More than half of all patients treated with ESC was diagnosed with a depression as primary diagnosis (*n* = 157, 52.3%). The remaining patients were either diagnosed with other diagnosis than depression (*n* = 92, 30.7%), or no information on diagnosis was available (*n* = 51, 17.0%). For about every second patient, only one diagnosis was noted on the request form (53%, *n* = 132 of 249). The other half of the patients was diagnosed with a minimum of one and up to ten additional comorbid psychiatric and/or somatic conditions (*n* = 117 of 249). From the sample of depressed patients (*n* = 157), 42.7% of patients had a comorbid psychiatric diagnosis (*n* = 67). Frequent additional comorbid diagnoses were alcohol- and substance-related disorders (ICD F10.2; F10.3 *n* = 36, ICD F13.2 *n* = 8), anxiety and related disorders (ICD F40, 41, 42, 43, *n* = 21) and personality disorders (ICD F60, *n* = 9). Patients without depression (*n* = 92) were either treated/cotreated with ESC for an anxiety or related disorder (37.0%, ICD F40/41/42/43), schizophrenic spectrum disorder (21.7%, ICD F20-F29), or bipolar disorder (15.2%, ICD F30/31). The reason for TDM was reported in 190 patients. In 43.2% of all requests, the reason for TDM was follow-up control. Additional reasons for TDM were start of medication (26.3%), compliance control (21.1%), change in medication (11.1%), nonresponse (3.7%) and side effects (1.6%). The majority included request forms had rather been a repeated measure of the drug level than first monitoring (18% “first”). Concomitant medication was frequent, and it was reported in 72.3% of patients with up to 10 additional drugs and 2.0 concomitant drugs on average (for full list, see Supplementary Table 1). Other CNS-relevant drugs were given in 187 of all patients. An additional antidepressant drug was given to 121 (40.3%) of them; most preferred was mirtazapine (68.6%), followed by trimipramine (10.7%). Overall, benzodiazepines were given in 26 patients in addition to their treatment with ESC. 83 patients were treated with ESC monotherapy.

For more than half of the patients, the CGI severity score was available (*n* = 165). Most patients were classified as markedly ill (CGI.S; 5, 32.1%, *n* = 53) and severely ill (CGI-S; 6, 40.6%, *n* = 67). The CGI-improvement score was noted for 154 patients with most patients classified as much improved (CGI-I; 2, 45.5%) or minimally improved (CGI-I; 3, 23.4%). Overall, the response rate was 59.5% in 163 patients for whom information on response was available. The UKU scale was available for 148 patients. 25 patients experienced side effects with most of them experiencing tension/inner restlessness (*n* = 18).

The mean (± SD) ESC dose was 16.9 mg ± 7.3 mg/day in all 300 patients. In total, most common doses were 10 mg (31.3%), 15 mg (17.3%) and 20 mg (36.0%). 3.3% of patients had doses lower than 10 mg, and 12% of patients were treated with doses above 20 mg. The mean serum concentration of ESC was 28.3 ± 20.6 ng/mL (2.5–105.0 ng/mL, *n* = 300), the mean serum concentration of D-ESC was 13.1 ± 9.5 ng/mL (2.5–78.0 ng/mL, *n* = 297), and the mean DD-ESC concentration was 4.5 ± 8.2 ng/mL (0.0–76.0, *n* = 83).

### Clinical effects for depressive patients treated with ESC

A total of 157 patients were treated with ESC for depressive disorder (ICD 10 F32/F33). Of those, the majority received additional CNS-relevant medications (*n* = 103). Detailed information on patients with depression with and without CNS-relevant comedication can be found in Table 1. Mean doses of patients treated with ESC for depression were 17.0 ± 6.8 mg/day (5–40). Mean ESC and S-DCT serum concentrations were 29.7 ± 21.0 ng/mL (median 23.0, IQR 16.0–41.5) and 13.4 ± 10.6 ng/mL (median 11.0, IQR 6.0–17.0). For the majority of patients with depression, ESC serum levels within the therapeutic reference range of 15–80 ng/mL were detected (73.3%, *n* = 115). 23.6% of patients had levels below and 3.2% of patients had concentrations above this range.

**Table 1:** Demographic data, CGI scores, daily doses and serum concentrations of escitalopram and its active metabolites in patients with major depression

Sample (*n*) (male/female/unknown)		300	(119/180/1)
Patients with depression (*n*) (male/female)		157	(55/102)
Patients with depression under S-CT monotherapy (*n*) (male/female) Patients with S-CT monotherapy (*n*) (male/female)		53 109	(19/34) (48/61)
Patients with depression (*n* = 157)			
Age, years	Mean ± SD (range)	52.6 ± 16.2	(18–86)
No. of Comedication	Mean ± SD	2.1 ± 2.1	
CGI severity score			
–of all depressive patients (*n* = 91)	Mean ± SD (range)	5.8 ± 1.0	(2–8)
–of all depressive patients under S-CT monotherapy (*n* = 26)	Mean ± SD (range)	5.8 ± 0.9	(4–8)
CGI-improvement score			
–of all depressive patients (*n* = 84)	Mean ± SD (range)	2.3 ± 1.0	(1–5)
–of all depressive patients under S-CT monotherapy (*n* = 23)	Mean ± SD (range)	2.1 ± 1.1	(1–5)
S-CT dose, mg/d			
–of all depressive patients (*n* = 157),	Mean ± SD (range)	17.0 ± 6.8	(5–40)
–of all depressive patients under S-CT monotherapy (*n* = 53), mean ± SD (range)	Mean ± SD (range)	15.5 ± 5.1	(5–25)
Serum concentrations, ng/mL			
–S-CT (*n* = 157)	Mean ± SD (range) Median (IQR)	29.7 ± 21.0 23.0	(2.5–99.0) (16.0–41.5)
–D-SCT (*n* = 156)	Mean ± SD (range) Median (IQR)	13.4 ± 10.6 11.0	(2.5–78.0) (6.0–17.0)
–DD-SCT (n = 46)	Mean ± SD (range) Median (IQR)	5.2 ± 11.0 2.5	(0.0–76.0) (2.5–5.0)
Metabolite-to-parent compound ratio (MPR)			
–D-SCT/S-CT) (*n* = 156) –DD-SCT/ S-CT) (*n* = 46)	Mean ± SD (range) Mean ± SD (range)	0.6 ± 0.4 0.2 ± 0.3	(0.1–2.2) (0.0–1.9)
Dose-corrected serum concentrations (C/D), ng/mL/mg			
–S-CT/Dose (*n* = 157) –D-SCT/Dose (*n* = 156)	Mean ± SD (range) Mean ± SD (range)	1.8 ± 1.2 0.8 ± 0.8	(0.3–6.8) (0.1–8.0)

Antidepressant efficacy of ESC alone was assessed in 51 patients independent from diagnosis (shown in supplemental Table II). ESC concentrations were higher in responders (median 17.0; *n* = 30) than in nonresponders (12.0; *n* = 21) (not significant). The ROC curve identified a cut-off point of 14.5 ng/mL that discriminates responders from nonresponders (AUC 0.652, *p* 0.066, shown in supplemental Fig. 2). 64.1% of patients with a drug level above 14.5 ng/mL responded to the ESC treatment. The response rate below this threshold was 41.7%. When selecting patients with ESC as the only antidepressant and without other CNS-relevant comedication, 50 patients with information on side effects were available. Specific side effects were reported in 12 patients. The most frequently reported side effect was tension/unrest in 8 cases. Their mean ESC BL was 36.0 ± 33.5, and the mean dose was 15.6 mg ± 5.0.

### Influencing factors on ESC, S-DCT and DS-DCT blood levels in patients treated with ESC

**Fig. 1:**
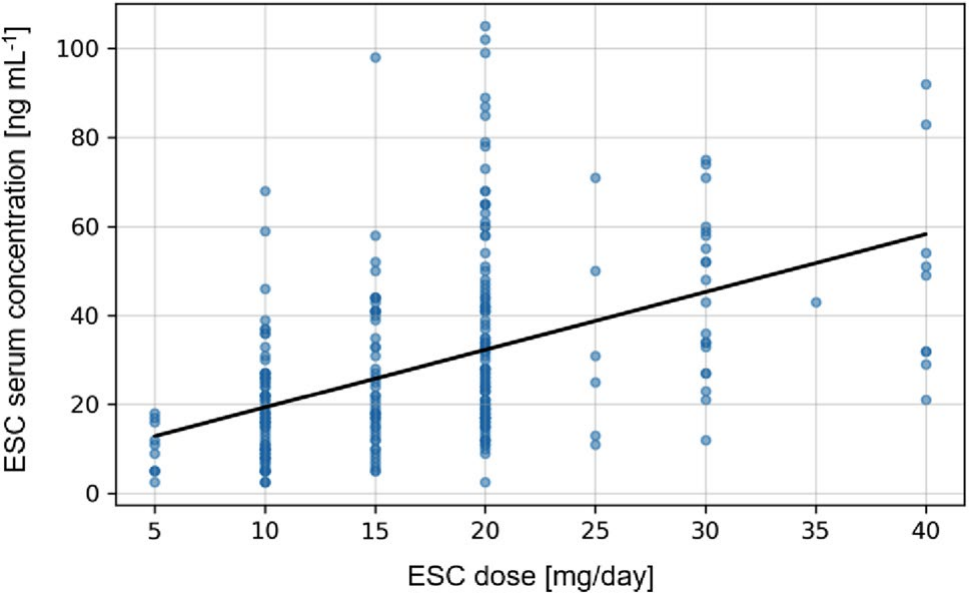
Linear regression of ESC dose and serum concentration (*n* = 300, *r* = 0.52; *P* < .0001)

The total sample showed a good correlation between BL and applied ESC doses (*n* = 300, *r* = 0.52; *P* < 0.0001), S-DCT (*n* = 297, *r* = 0.63; *P* < 0.0001) and DD-ESC (*n* = 83, *r* = 0.26, *p* = 0.0018). Figure 1 illustrates a high inter-individual variation in ESC BLs among all dosage levels. Mean C/D ratios and MPRs for men and women and for different age groups are presented in Table 2. C/D ratios of the total sample were 1.72 ± 1.11 for ESC and 0.79 ± 0.61 for S-DCT. Mean MPRs were 0.58 ± 0.36 and 0.21 ± 0.25 for D-ESC/ESC and DD-ESC/ ESC.

**Table 2:** Metabolic ratios in men and women and different age groups (Mann–Whitney/Kruskal–Wallis)

	C/D S-CT ng/ml/mg	*n*	C/D D-SCT ng/ml/mg	*n*	MPR (D-SCT/S-CT)	*n*	MPR (DD-SCT/S-CT)	*n*
Male	**1.54 ± 0.94**	119	**0.71 ± 0.39**	119	0.58 ± 0.34	119	0.19 ± 0.16	34
Female	**1.84 ± 1.19**	180	**0.84 ± 0.72**	177	0.58 ± 0.38	177	0.22 ± 0.30	49
< 20	**1.73 ± 0.96**	10	**1.37 ± 2.28**	10	0.71 ± 0.61	10	NA	0
20–29	**1.85 ± 1.05**	30	**0.71 ± 0.36**	30	0.48 ± 0.27	30	0.11 ± 0.06	11
30–39	**1.47 ± 0.76**	52	**0.78 ± 0.40**	52	0.64 ± 0.38	52	0.19 ± 0.16	12
40–49	**1.46 ± 0.97**	74	**0.67 ± 0.33**	73	0.63 ± 0.43	73	0.21 ± 0.15	26
50–59	**1.79 ± 1.28**	55	**0.79 ± 0.34**	54	0.61 ± 0.34	54	0.24 ± 0.43	18
60–69	**1.89 ± 1.23**	40	**0.79 ± 0.81**	39	0.46 ± 0.29	39	0.28 ± 0.28	13
70–79	**1.98 ± 1.08**	29	**0.92 ± 0.37**	29	0.55 ± 0.23	29	0.07	2
> 80	**2.85 ± 1.7**	9	**0.98 ± 0.54**	9	0.41 ± 0.24	9	0.07	1
Total	1.72 ± 1.11	299	0.79 ± 0.61	296	0.58 ± 0.36	296	0.21 ± 0.25	83

Significant differences between groups in bold

C/D ratios between gender groups (S-CT *p* .03, D-SCT *p* .02) and age groups (S-CT *p* .024, D-SCT *p* .031). MPRs between gender groups (D-SCT/S-CT *p* .70, DD-SCT/S- CT *p* .75) and age groups (D-SCT/S-CT *p* .13, DD-SCT/S-CT *p* .37)

ESC and D-ESC BLs showed a good correlation with sex (*n* = 299, *r* = 0.16, *p* = 0.006 and *n* = 296, *r* = 0.15, *p* = 0.010). This correlation could not be observed for DD-ESC drug levels (*n* = 83). Men showed 20% (C/D; 1.54, *n* = 119) lower dose-corrected concentrations than woman (C/D; 1.84, *n* = 180). This difference was statistically significant, also for the metabolite (*p* = 0.03 and *p* = 0.010). As a consequence, men in general had lower mean ESC and D-ESC concentrations compared to women (23.6 ± 15.5 and 31.5 ± 22.9 ng/mL, *p =* 0.006; 11.4 ± 7.6 and 14.2 ± 10.5 ng/mL, *p =* 0.012).

Furthermore, age positively correlated with the ESC concentration (*n* = 299, *r* = 0.11, *p* = 0.05) and with the dose-corrected ESC (*n* = 299, *r* = 0.130, *p* = 0.025) and D-ESC concentrations (*n* = 296, *r* = 0.124, *p* = 0.033). Drug levels increased with age, especially in patients 60 years and older.

A multivariate regression analysis using threefold cross-validation (permutated 1000 times, *n* = 299) was performed including ESC concentrations and the variables dose, age and sex. The accuracy of the prediction was averaged over all cross-validations and permutations. Based on dose, age and sex, the models could predict ESC BLs with an average generalized coefficient of determination of D^2^ = 0.22 ± 0.059 (shown in supplemental Table II).

### Influences of pathological liver alterations, alcohol and benzodiazepine dependence

Laboratory markers GGT and the AST/ALT values were available for 68 patients (50% of them diagnosed with a depression). Of these, patients were classified as patients with liver dysfunctions indicated by elevated GGT and AST or ALT with AST/ALT ratio ≥1. 15 patients had an elevated GGT with an AST/ALT ratio ≥1 without regard to single AST/ALT values. In this group, dose-corrected ESC concentrations were higher compared to patients without (*n* = 51) liver dysfunctions identified by GGT and AST/ALT ratio (*p* = 0.013; mean 2.23 ± 0.27 vs. 1.51 ± 0.12 ng/mL/ mg/day). Of all patients in which the liver values were available, 39 and 33% of patients with alcohol or benzodiazepine dependence showed abnormal GGT and AST/ALT ratio that have in this study been identified an influence on ESC BLs. C/D ratios and MPRs did not considerably vary in patients with alcohol dependence and patients without this diagnosis (shown in Table 3).

**Table 3:** Metabolic ratios in patients with alcohol (F10) or substance use disorder (F13) and under benzodiazepine use compared to control group (Mann–Whitney/Kruskal–Wallis)

	C/D-ESC ng/ml/mg	*n*	C/D D-ESC ng/ml/mg	*n*	MPR (D-ESC/ESC)	*n*	MPR (DD-ESC/ESC)	*n*
Alcohol use disorder (F10)	1.73 ± 1.11	68	0.78 ± 0.36	67	0.61 ± 0.40	67	0.19 ± 0.15	39
Substance use disorder (F13)	1.88 ± 1.35	15	0.78 ± 0.61	15	0.58 ± 0.37	15	0.20 ± 0.26	9
Benzodiazepine use	1.87 ± 1.09	26	**0.89 ± 0.29**	26	0.59 ± 0.26	26	0.54 ± 0.88	4
Liver abnormalities from lab results and sonography	1.56 ± 0.68	8	0.77 ± 0.31	7	0.57 ± 0.22	7	0.27 ± 0.14	5

Significant differences between groups in bold

When compared to a control group, a higher number of men constituted the patient group suffering from alcohol use disorder (shown in Table 4). Patients with the disorder were less severely ill (CGI-S), and they were treated with lower doses resulting in lower ESC concentrations. The interquartile concentration range was 11–34 ng/mL. 69.1% of patients had concentrations within the recommended reference range for antidepressant treatment with ESC, and 30.9% had concentrations below this range. No concentration above this range was detected. With comparable doses, patients with acute benzodiazepine use (*n* = 26) and patients with benzodiazepine use disorder (*n* = 15) showed a trend towards higher dose-corrected concentrations compared to patients without these disorders. This effect did, however, only reach significance for D-ESC in the subgroup with acute benzodiazepine use (*p =* 0.009). Of note, a small sample of patients with documented liver abnormalities confirmed by sonography (e.g. K76.0, K70.0) had lower dose-corrected concentrations compared to controls (*n* = 8, C/D 1.56 ± 0.68, not significant).

**Table 4:** Patients with and without alcohol use disorder (F10) (Mann–Whitney/Kruskal–Wallis)

	Patients with alcohol use disorder	Patients without alcohol use disorder	Total	p value
Sample size	68	179	247	
–with depression	37 (54.5%)	120 (67%)	157 (63.6%)	
Age (years)	47.6 ± 10.4	50.5 ± 17.7	49.7 ± 16.0	0.207
Sex % female	**44.1%**	**63.1%**	**60.2%**	**0.007**
CGI-S	**5.3 ± 0.82**	**5.8 ± 0.95**	**5.7 ± 0.94**	**0.016**
Dose (mg/day)	**14.85 ± 6.6**	**17.5 ± 7.2**	**16.7 ± 7.1**	**0.007**
S-CT concentration (ng/mL)	23.8 ± 14,9	29.8 ± 21,9	28.1 ± 20.3	0.131
S-DCT concentration (ng/mL)	11.4 ± 7.0	13.9 ± 10.7	13.3 ± 9.9	0.197
C/D ratio	1.73 ± 1.11	1.71 ± 1,13	1.72 ± 1.12	0.916
MPR D-SCT/S-CT	0.61 ± 0.40	0.57 ± 0.35	0.58 ± 0.36	0.759

Significant differences between groups in bold

## Discussion

This study presents an overview of the treatment effects and pharmacokinetics of ESC in patients treated in a naturalistic setting, including the interaction potential of comorbidities such as alcohol and substance use disorders. An optimal antidepressant effect for ESC is expected within a recommended target range of 15–80 ng/mL [15]. The majority of our patients (72%) had serum concentrations within this range, and they were treated within the approved dosage range of 10–20 mg. However, 11% of patients required doses above 20 mg to reach drug levels within the recommended therapeutic reference range. More concerning is that every fourth patient (25.6%) treated with an approved dosage did not reach the target threshold concentration of 15 ng/mL. The results of our efficacy analysis confirm the recommended threshold of 15 ng/mL, above which antidepressant response becomes more likely, in a sample of patients treated with ESC monotherapy [15]. The interquartile range from patients with depression was 16.0–41.5 ng/mL, and with 13.5–25.3 ng/mL it was somewhat lower in responders. The overall response rate of 59.5% was in line with previous studies [22]. The majority of samples included in this study were follow-up measurements. As an explanation for follow-up concentrations below the therapeutic reference range, placebo response under antidepressant drug treatment has been frequently discussed in drug monitoring trials [23].

Patients with alcohol use disorders were prescribed lower ESC doses, resulting in lower drug concentrations. Less severe depressive symptoms (according to CGI-S) in this population might have led to prescription of lower ESC doses. However, a different response pattern to antidepressant treatment in patients with alcohol use disorder remains a possibility. Patients with alcohol dependence did not show considerably differing metabolic ratios compared to patients without this comorbidity. An effect on drug levels could more likely be explained by other factors such as female sex, higher age and liver dysfunction. The relationship of applied doses, age and sex with the ESC serum concentrations could be partially described by a linear function. However, most of the variation of ESC serum concentrations could not be predicted by these variables and, thus, highlights the necessity of clinical measurements of serum concentrations in case of insufficient response.

Reasons why gender may affect pharmacokinetics are molecular as well as physiological factors. Men are supposed to have a higher activity of CYP1A2, P-glycoprotein and some isoforms of glucuronosyltransferases and sulfotransferases. In women, CYP2D6 activity is higher. Physiological factors are women’s generally lower body weight and organ size, higher percentage of body fat, lower glomerular filtration rate and different involvement of steroid hormones that may influence the activity of all three CYP isoenzymes metabolizing ESC and citalopram [24]. The univariate correlation of sex and ESC serum concentration can be attributed to multifarious potential covariates such as body weight, body composition or metabolic properties. In our study, mean serum concentration and C/D ratio were in line with values previously reported [13, 25, 26], however, higher than those indicated in the TDM guidelines [15]. Our findings confirm the results of Waade et al., 2014 [7], who reported 15% lower metabolic ratios in women compared to men. In line with other studies, we found increasing C/D ratios with age [2].

The results of this study should be interpreted cautiously. First, the routine TDM setting did not allow us to control patient adherence to the treatment, nor to control for other influences on antidepressant responses. Not only psychological interventions (e.g. psychotherapy) and psychosocial factors (e.g. stress levels and social support), but also a series of other factors like hypothyroidism, hormonal changes, nutrition deficiencies, or sleep disorders (e.g. insomnia and obstructive sleep apnoea) might be relevant in this context.

Second, the patients included in the study were not genotyped, altered C/D ratios may be a result of CYP2D6 and CYP2C19 genetic variability. The activity of both isoenzymes is of major importance in the biotransformation of ESC and many other drugs. The relatively high extent of polypharmacy of on average two co-administered drugs may have contributed to this effect. Co-prescription of potent CYP2D6 inhibitors, CYP3A4 inhibitors/inducers or CYP2C19 inhibitors/inducers was identified from the requisition forms. Since less than 2% of patients per group were co-administered with relevant comedication, the effects of comedication were considered negligible. However, a potential influence of comedication cannot be ruled out, especially in subgroups of older patients with increasing polypharmacy.

The diagnosis of alcohol or benzodiazepine dependence alone may not affect ESC BLs, but liver dysfunction does. Reduced liver function, indicated by elevated GGT and AST/ALT ratio, resulted in higher dose-corrected ESC concentrations. Previous studies could not find clinically relevant differences in ESC, D-ESC and DD-ESC levels in patients with hepatic impairment compared to healthy adults [27].

To sum up, the present study strongly supports a target concentration of 15 ng/mL for antidepressant response. 75% of all patients with depression had BLs below 42 ng/mL. Patients with comorbid alcohol use disorder in treatment might require even lower concentrations (interquartile concentration 11–34 ng/mL). Clearly, further prospective studies are needed to confirm our findings.

## Conclusion

This study adds evidence to the results from previous studies indicating that age, sex and liver dysfunction affect the serum levels of ESC and its metabolite D-ESC. Pronounced pharmacokinetic variability requires dosages above the approved maximum daily dosage in a relevant number of patients and supports the level 2 (“recommended”) recommendation of the AGNP expert group [15] to monitor ESC serum levels for treatment optimization.

## Supplementary Information

Below is the link to the electronic supplementary material.

Supplementary file1 (DOCX 180 KB)

## Data Availability

The original contributions presented in the study are included in the article, and further inquiries can be directed to the corresponding author.

## References

[1] Schwabe U, Ludwig W-D. Arzneiverordnungs-Report 2020

[2] Reis M, Chermá MD, Carlsson B, Bengtsson F (2007). Therapeutic drug monitoring of escitalopram in an outpatient setting. Ther Drug Monit.

[3] Reis M, Aamo T, Spigset O, Ahlner J (2009). Serum concentrations of antidepressant drugs in a naturalistic setting: compilation based on a large therapeutic drug monitoring database. Ther Drug Monit.

[4] Tsuchimine S, Ochi S, Tajiri M, Suzuki Y, Sugawara N, Inoue Y (2018). Effects of Cytochrome P450 (CYP) 2C19 Genotypes on Steady-State Plasma Concentrations of Escitalopram and its Desmethyl Metabolite in Japanese Patients With Depression. Ther Drug Monit.

[5] Unterecker S, Riederer P, Proft F, Maloney J, Deckert J, Pfuhlmann B (2013). Effects of gender and age on serum concentrations of antidepressants under naturalistic conditions. J Neural Transm (Vienna).

[6] Lundbeck Canada Inc. Product Monograph including patient medication information. Available from: https://www.lundbeck.com/content/dam/lundbeck-com/americas/canada/products/files/cipralex_product_monograph_english.pdf. [Accessed 18.02.2022]

[7] Waade RB, Hermann M, Moe HL, Molden E (2014). Impact of age on serum concentrations of venlafaxine and escitalopram in different CYP2D6 and CYP2C19 genotype subgroups. Eur J Clin Pharmacol.

[8] Jukić MM, Haslemo T, Molden E, Ingelman-Sundberg M (2018). Impact of CYP2C19 genotype on escitalopram exposure and therapeutic failure: a retrospective study based on 2,087 patients. Am J Psychiatry.

[9] Scherf-Clavel M, Deckert J, Menke A, Unterecker S (2019). Smoking Is associated with lower dose-corrected serum concentrations of escitalopram. J Clin Psychopharmacol.

[10] Rudberg I, Hendset M, Uthus LH, Molden E, Refsum H (2006). Heterozygous mutation in CYP2C19 significantly increases the concentration/dose ratio of racemic citalopram and escitalopram (S-citalopram). Ther Drug Monit.

[11] Rudberg I, Mohebi B, Hermann M, Refsum H, Molden E (2008). Impact of the ultrarapid CYP2C19*17 allele on serum concentration of escitalopram in psychiatric patients. Clin Pharmacol Ther.

[12] Florio V, Porcelli S, Saria A, Serretti A, Conca A (2017). Escitalopram plasma levels and antidepressant response. Eur Neuropsychopharmacol : J European Coll Neuropsychopharmacol.

[13] Leuchter AF, Cook IA, Marangell LB, Gilmer WS, Burgoyne KS, Howland RH (2009). Comparative effectiveness of biomarkers and clinical indicators for predicting outcomes of SSRI treatment in major depressive disorder: results of the BRITE-MD study. Psychiatry Res.

[14] Lloret-Linares C, Bosilkovska M, Daali Y, Gex-Fabry M, Heron K, Bancila V (2018). Phenotypic assessment of drug metabolic pathways and P-glycoprotein in patients treated with antidepressants in an ambulatory setting. J Clini Psychiatry..

[15] Hiemke C, Bergemann N, Clement HW, Conca A, Deckert J, Domschke K (2018). Consensus guidelines for therapeutic drug monitoring in neuropsychopharmacology: update 2017. Pharmacopsychiatry.

[16] World Health Organization. International Statistical Classification of Diseases and Related Health Problems. 10th ed. Available online at: https://icd.who.int/browse10/2010/en (accessed July 13 2022)

[17] Lingjaerde O, Ahlfors UG, Bech P, Dencker SJ, Elgen K (1987). The UKU side effect rating scale. A new comprehensive rating scale for psychotropic drugs and a cross-sectional study of side effects in neuroleptic-treated patients. Acta Psychiatr Scand Suppl.

[18] Guy W. ECDEU assessment manual for psychopharmacology. Rockville, Md.: U.S. Dept. of Health, Education, and Welfare, Public Health Service, Alcohol, Drug Abuse, and Mental Health Administration, National Institute of Mental Health, Psychopharmacology Research Branch, Division of Extramural Research Programs. 1976

[19] Shams M, Hiemke C, Härtter S (2004). Therapeutic drug monitoring of the antidepressant mirtazapine and its Ndemethylatedmetabolite in human serum. Ther Drug Monit.

[20] Baral N, Pokhrel S, Lamsal M, Yadav BN, Sah SP (2005). Utility of gamma-glutamyl transpeptidase and mean corpuscular volume in alcoholic liver disease. Southeast Asian J Trop Med Public Health.

[21] Pedregosa F, Varoquaux G, Gramfort A, Michel V, Thirion B, Grisel O, et al. 2012 Scikit-learn: Machine Learning in Python. Journal of Machine Learning Research. 12

[22] Yang LP, Scott LJ (2010). Escitalopram: in the treatment of major depressive disorder in adolescent patients. Paediatr Drugs.

[23] Hiemke C (2019). Concentration-effect relationships of psychoactive drugs and the problem to calculate therapeutic reference ranges. Ther Drug Monit.

[24] Meibohm B, Beierle I, Derendorf H (2002). How important are gender differences in pharmacokinetics?. Clin Pharmacokinet.

[25] Engelmann J, Wagner S, Solheid A, Herzog DP, Dreimüller N, Müller MB (2021). Tolerability of high-dose venlafaxine after switch from escitalopram in nonresponding patients with major depressive disorder. J Clin Psychopharmacol.

[26] Florio V, Porcelli S, Saria A, Serretti A, Conca A (2017). Escitalopram plasma levels and antidepressant response. Eur Neuropsychopharmacol.

[27] Rao N (2007). The clinical pharmacokinetics of escitalopram. Clin Pharmacokinet.

