# Supplementary material for: Concentrations of escitalopram in blood of patients treated in a naturalistic setting: focus on patients with alcohol and benzodiazepine use disorder
Source: Eur Arch Psychiatry Clin Neurosci. 2022 Oct 7;273(1):75–83. doi: 10.1007/s00406-022-01491-9 (PMC9958172; doi:10.1007/s00406-022-01491-9)
Supplement: Supplementary file 1 — Supplementary file1 (DOCX 180 KB) [file 406_2022_1491_MOESM1_ESM.docx]

***Supplementary material: “Concentrations of escitalopram in blood of patients treated in a naturalistic setting: Focus on patients with alcohol and benzodiazepine use disorder”***

Supplemental Figure 1. Gamma distribution approximating the histogram of escitalopram plasma concentrations


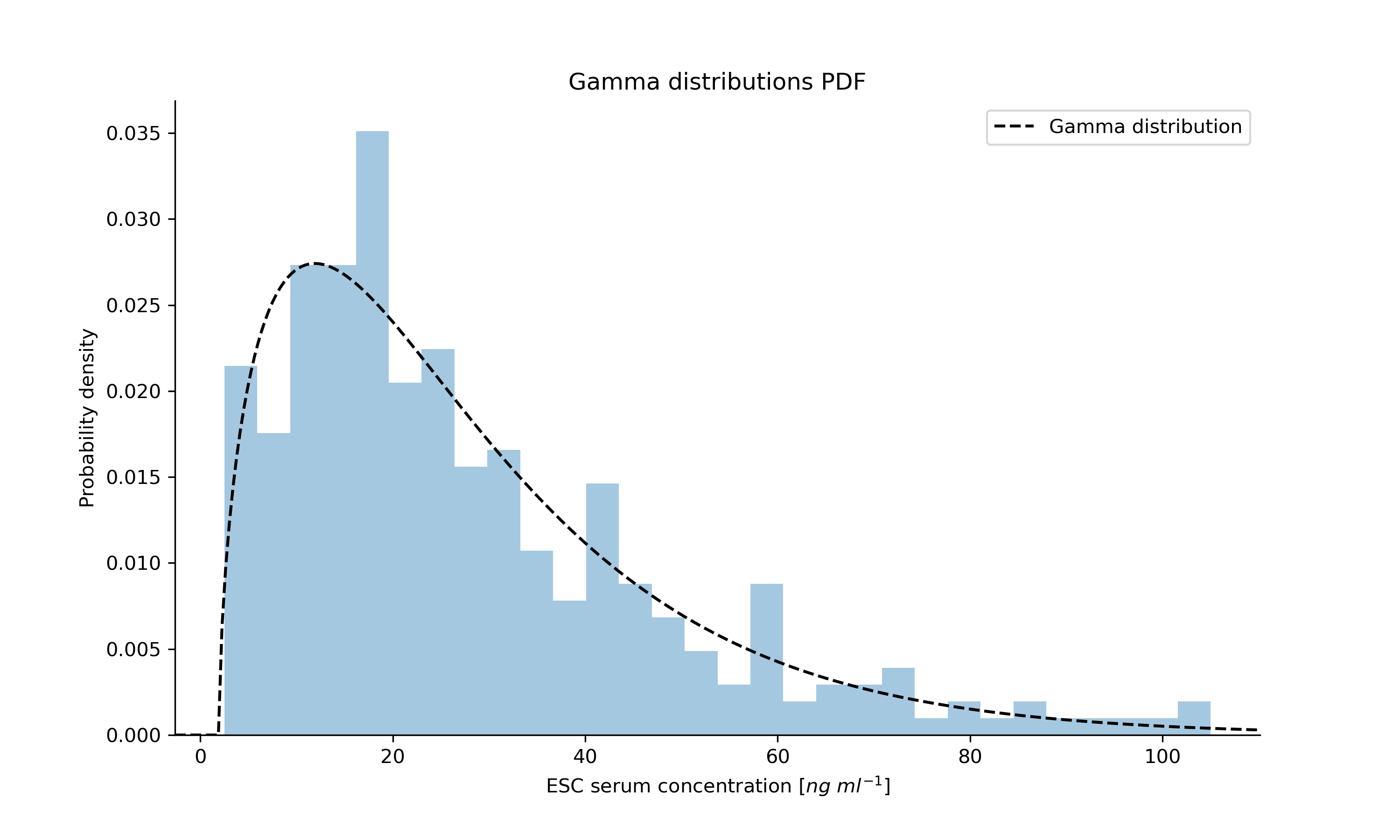


Supplemental Table 1. Model parameters for dataset consisting of all 301 subjects using 3-fold cross-validation, the cross-validation was permutated 1000 times.

| **Variable** | **Mean estimate** | **SD of mean estimate** |
| --- | --- | --- |
| Intercept | -0.622 | 0.051 |
| Dose | 0.048 | 0.004 |
| Age | 0.005 | 0.001 |
| Sex | 0.191 | 0.052 |
| **GENERALIED COEFFICIENT OF DETERMINATION** | **MEAN ESTIMATE** | **STANDARD DEVIATION OF MEAN ESTIMATE** |
| **D^2^** | 0.22 | 0.059 |

Additional information on the Generalized linear model

GLMs represent a class of regression models that model the relationship of predictor variables Xi,..,n and a response variable Y. The GLM is related to the response variable Y via a linear link function. The distribution of the response variable Y is assumed to be a gamma distribution.

We used a GLM to predict the escitalopram plasma concentration. First modelling approaches predicted the escitalopram plasma concentration based on various pharmacokinetic predictor variables (see results). Due to sparse data availability of weight and sparse data availability of cytochrom activity altering comedication, all modelling attempts yielded inferior results with a D2 < 0.2 indicating over-fitting due to the relatively small sample size in the cross-validation step. Thus, data with less than 10% entries in the global dataset were excluded and dosage of escitalopram, age and sex were selected as predictors for the GLM. Modelling approaches deploying linear models with other link functions or distributions as well as Bayesian ridge regression yielded inferior results (not depicted).

*Supplemental Figure 2. ROC curve for 51 patients under S-CT monotherapy. AUC 0.652*


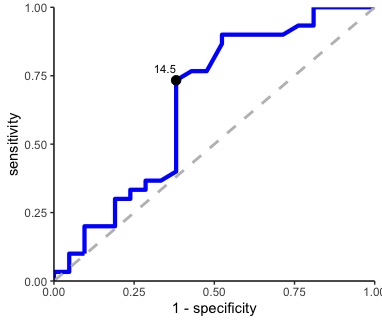


Supplemental Table 2. *Patients treated with S-CT monotherapy independent from diagnosis.*

|  | S-CT Responders | S-CT Nonresponders | All |  |
| --- | --- | --- | --- | --- |
| Sample size | 30 | 21 | 51 |  |
| Age (years) | 43.1 ± 10.4 | 50.1 ± 19.0 | 45.9 ± 14.8 | .119 |
| Sex male/female | 14/16 | 11/10 | 25/26 |  |
| Dose (mg) | 14.7 ± 5.7 | 14.5 ± 7.7 | 14.6 ± 6.6 | .659 |
| S-CT concentration (ng/mL)  Median (IQR) | 23.1 ± 17.2  17.0 (13.5-25.3) | 18.2 ± 16.0  12.0 (8.5-22.5) | 21.1 ± 16.8  16.0 (10.0-25.0) | .066 |
| D-S-CT concentration (ng/mL)  Median (IQR) (n=50) | 11.0 ± 7.7 9.0  9 (5.0-15.5) n=29 | 12.4 ± 13.1  6 (5.0-15.0) | 11.6 ± 10.2  8.0 (5.0-15.3) | .50 |
| C/D ratio | 1.73 ± 1.23 | 1.35 ± 1.34 | 1.57 ± 1.28 | .55 |
| MPR (n=50) | 0.63 ± 0.42 | 0.70 ± 0.30 | 0.66 ± 0.37 | .11 |

Note: Data are expressed as mean ± standard deviation unless otherwise specified

*List of coadministered medication at the time of blood sampling, available for 218 subjects*

|  | **Coadministered drugs** |
| --- | --- |
| 1 | Acetazolamid |
| 2 | Alprazolam |
| **3** | Amisulprid (abgesetzt) |
| 4 | Amisulprid,Clozapin |
| 5 | Amisulprid,Mirtazapin |
| 6 | Amlodipin,Ramipril,ASS,Pantaprazol,Furosemid |
| 7 | Aripiprazol |
| 8 | Aspirin,Lorazepam,Nitrazepam,Olanzapin |
| 9 | ASS 100,Bisoprolol,simvastatin,Pipamperon,Voltaren,Minobal?,Mirtazapin |
| 10 | ASS 100,Pantaprazol,Dutasterid,Lithium,Quetiapin |
| 11 | ASS,Durapindol,Reboxetin,Pindolol |
| 12 | ASS,Mirtazapin,Gabapentin |
| 13 | Atorvastatin,Clopidogrel |
| 14 | Atorvastatin,Mirtazapin |
| 15 | Bisoprolol |
| 16 | Bisoprolol,Candesartan,Pantaprazol,ASS 100,Olanzapin,Magnesium,Lorazepam |
| 17 | Bisoprolol,HCT,Atenolol,Allopurinol,Ibuprofen,Simvastatin |
| 18 | Cabergolin |
| 19 | Candesartan Plus,Valproat Chrono |
| 20 | Carbamazepin |
| 21 | Carbamazepin ret. |
| 22 | Carbamazepin,Metoprolol |
| 23 | Carbamazepin,Risperidon |
| 24 | Cineol (Soledum),levofloxacin,mometasone furoate spray,oxymetazolin Spray,Budesonid,Reboxetin |
| 26 | Clomipramin,Venlafaxin |
| 27 | Clonazepam,Mirtazapin |
| 28 | Clozapin |
| 29 | Clozapin,Benserazide,Tamsulosin,Mirtazapin |
| 30 | Clozapin,Pipamperon |
| 31 | Clozapin,Prothipendyl |
| 32 | Cyclosporine,Magnesium,Mirtazapin |
| 33 | Diazepam,Lansoprazol,Mirtazapin |
| 34 | Diazepam,Melperon 125mg,Fluvastatin,Valproat |
| 35 | Diazepam,Mirtazapin,Quetiapin,Metoprolol |
| 36 | Diazepam,Risperidon,Presomen,Benalapril,ASS,Movicol,Budesonid, Formoterol,Biotin,Mirtazapin |
| 37 | Digoxin,Pantaprazol,Folsan,Atorvastatin,Verapamil mite,Mirtazapin 30mg |
| 38 | Dimenhydrinat,Lamotrigin,Reboxetin,Agomelatin,Melperon,Zolpidem,Acetylcholinesterase,Pantoprozol |
| 39 | Doxepin |
| 40 | Doxepin-HCl |
| 41 | Doxepin-HCl |
| 42 | Duloxetin |
| 43 | Eisen, Folsäure,Mirtazapin,Metoclopramid |
| 44 | Flupirtin,Trazodon |
| 45 | Flurazepam,Aspirin,Candesartan,Rofecoxib |
| 46 | Folsan,Pantaprazol,Trimipramin |
| 47 | Folsan,Vitamin B6 und B1,Furosemid |
| 48 | Gelomyrtol |
| 49 | Gelomyrtol,Quetiapin |
| 50 | Haloperidol |
| 51 | Hydrochlorothiazid,Metoprolol mite,ASS,Diazepam-Trpf,Pravastatin |
| 52 | Imipramin |
| 53 | Irbesartan,Verapramil,Folsäure,L-Thyroxin |
| 54 | Jodetten,Pantaprazol,Magaldrat,Quetiapin |
| 55 | Jodid |
| 56 | Kalium Brause,Magnesium-Verla,Allopurinol,Trimipramin |
| 57 | L-Thyroxin |
| 58 | L-Thyroxin |
| 59 | L-Thyroxin,Amitriptylin ret.,Risperidon |
| 60 | L-Thyroxin,Calcium, Vitamin D3 |
| 61 | L-Thyroxin,Ginkgo,Acetylcystein long,Olanzapin |
| 62 | L-Thyroxin,Lisinopril,Hydrochlorothiazid,Amlodipin,Ramipril,Lithium ret.,Clozapin |
| 63 | L-Thyroxin,Mirtazapin |
| 64 | L-Thyroxin,Mirtazapin |
| 65 | L-Thyroxin,Propranolol,Lorazepam,Perazin,Prothipendyl,Aripiprazol, |
| 66 | L-Thyroxin,Ramipril,Zolpidem,Mirtazapin,Ibuprofen |
| 67 | L-Thyroxin,Trimipramin |
| 68 | Lactulose,Reboxetin,Olanzapin,Lithium,Lamotrigin,Mirtazapin |
| 69 | Lamotrigin |
| 70 | Lamotrigin,Olanzapin,Clomipramin,Lithium ret.,L-Thyroxin,Lisinopril,Zolpidem,Mirtazapin |
| 71 | Lisinopril,ASS,Simvastatin forte,L-Thyroxin,Olanzapin |
| 72 | Lithium ret. |
| 73 | Lithium ret.,Quetiapin,Reboxetin |
| 74 | Lithium, Reboxetin |
| 75 | Lorazepam,Amisulprid |
| 76 | Lorazepam,Hydrochlorothiazid,Atorvastatin,Pyridoxin,Amisulprid |
| 77 | Lorazepam,Mirtazapin |
| 78 | Lorazepam,Olmesartan,Lercanidipin,Glibenclamid,Pioglitazone,Metformin,Mirtazapin |
| 79 | Lorazepam,Pantaprazol,Nilvadipin |
| 80 | Lorazepam,Prothipendyl,Melperon,Amisulprid |
| 81 | Magnesium-Verla,Kalium,Carbamazepin,Prednisolon,Pantaprazol,L -Thyrox |
| 82 | Magnesium-Verla,Slow-Sodium,Mirtazapin |
| 83 | Magnesium,Benalapril,Natriumchlorid |
| 84 | Magnesium,Verapamil,Prednisolon,Pantaprazol,Lactulose, Eisen, Folsäure,Vitamin D3,Vitamin K |
| 85 | Metformin,Atorvastatin,L-Thyroxin,Ramipril,Pantaprazol,ASS |
| 86 | Metoprolol |
| 87 | Metoprolol |
| 88 | Metoprolol |
| 89 | Metoprolol-mite,Atorvastatin,Doxepin,Captopirl |
| 90 | Metoprolol,Benalapril,Hydrochlorothiazid,Pantaprazol,Acamprosat,Insulin |
| 91 | Metoprolol,Hydrochlorothiazid,Triamteren,Trimipramin,Mirtazapin,Melatonin,Diclofenac,Metoprolol |
| 92 | Mirtazapin |
| 93 | Mirtazapin |
| 94 | Mirtazapin |
| 95 | Mirtazapin |
| 96 | Mirtazapin |
| 97 | Mirtazapin |
| 98 | Mirtazapin |
| 99 | Mirtazapin |
| 100 | Mirtazapin |
| 101 | Mirtazapin |
| 102 | Mirtazapin |
| 103 | Mirtazapin |
| 104 | Mirtazapin |
| 105 | Mirtazapin |
| 106 | Mirtazapin |
| 107 | Mirtazapin |
| 108 | Mirtazapin |
| 109 | Mirtazapin |
| 110 | Mirtazapin |
| 111 | Mirtazapin |
| 112 | Mirtazapin |
| 113 | Mirtazapin |
| 114 | Mirtazapin |
| 115 | Mirtazapin |
| 116 | Mirtazapin |
| 117 | Mirtazapin |
| 118 | Mirtazapin |
| 119 | Mirtazapin |
| 120 | Mirtazapin |
| 121 | Mirtazapin |
| 122 | Mirtazapin |
| 123 | Mirtazapin |
| 124 | Mirtazapin |
| 125 | Mirtazapin,Jodetten,Jodthyrox,Sulpirid |
| 126 | Mirtazapin,Lamatrigin |
| 127 | Mirtazapin,Lamotrigin |
| 128 | Mirtazapin,Lorazepam,Amisulprid,Mirtazapin |
| 129 | Mirtazapin,Lorazepam,Lamotrigin,Olanzapin,Pyridoxin,Orale Kontrazeptiva |
| 130 | Mirtazapin,Metoprolol mite,Amitriptylin ret. |
| 131 | Mirtazapin,Nebivolol-HCl |
| 132 | Mirtazapin,Olanzapin,Ramipril,Digitoxin,Metoprolol mite,Triamteren, Hydrochlorothiazid,Mirtazapin |
| 133 | Mirtazapin,Quetiapin |
| 134 | Mirtazapin,Ramipril,Nortriptylin,Aspirin |
| 135 | Naltrexon |
| 136 | Naltrexon,Promethazin,Lorazepam,Olanzapin |
| 137 | Nebivolol,Jodetten,Mirtazapin |
| 138 | Nifedipin,Mirtazapin,Atenolol |
| 139 | Olanzapin |
| 140 | Olanzapin |
| 141 | Olanzapin |
| 142 | Olanzapin |
| 143 | Olanzapin |
| 144 | Olanzapin 10mg,Pipamperon 40mg,Haloperidol 5mg,Eisen,Vitamin B6 und B1 ,Propranolol,Xylometazolin NS,Pantaprazol, Lithium |
| 145 | Olanzapin,Aripiprazol |
| 146 | Olanzapin,Eisen |
| 147 | Olanzapin,L-Thyroxin |
| 148 | Olanzapin,Lamotrigin |
| 149 | Olanzapin,Lithium ret.,Ciprofloxacin,Mirtazapin |
| 150 | Olanzapin,Lorazepam,Lithium,L-Thyroxin,Hydrochlorothiazid,Zopiclon,Lisinopril,Mirtazapin |
| 151 | Olanzapin,Mirtazapin |
| 152 | Olanzapin,Mirtazapin |
| 153 | Olanzapin,Mirtazapin |
| 154 | Olanzapin,Mirtazapin |
| 155 | Olanzapin,Perazin |
| 156 | Olanzapin,Reboxetin,Mirtazapin |
| 157 | Orfiril,Pantaprazol,Diclofenac,Risperidon |
| 158 | Oxcarbazepin,Quetiapin |
| 159 | Pantaprazol,Amisulprid,Clozapin |
| 160 | Pantaprazol,Doxepin,Vitamin D3,Amitriptylin |
| 161 | Pantaprazol,Magnesium-Verla,Mirtazapin |
| 162 | Pantaprazol,Melatonin,Fraxiparin,Amitriptylin |
| 163 | Pantaprazol,Mirtazapin |
| 164 | Pantaprazol,N-Acetylcystein,Magnesium,Quetiapin |
| 165 | Pantaprazol,Olanzapin,Ziprasidon,Levomepromazin |
| 166 | Pantaprazol,Orale Kontrazeptiva |
| 167 | Pantaprazol,Quetiapin,Carbamazepin |
| 168 | Pantaprazol,Reboxetin |
| 169 | Pantaprazol,Trimipramin |
| 170 | Pantaprazol,Trimipramin,Lamotrigin |
| 171 | Paracetamol |
| 172 | Phenprocoumon |
| 173 | Pipamperon |
| 174 | Pipamperon,Amitriptylin,Alprazolam |
| 175 | Primidon,Promethazin |
| 176 | Prothipendyl,L-Thyroxin,Benazepril, Hydrochlorothiazid |
| 177 | Quetiapin |
| 178 | Quetiapin |
| 179 | Quetiapin |
| 180 | Quetiapin |
| 181 | Quetiapin |
| 182 | Quetiapin |
| 183 | Quetiapin |
| 184 | Quetiapin,Aripiprazol |
| 185 | Quetiapin,Carbamazepin ret.,Levomepromazin |
| 186 | Quetiapin,Eisen, Folsäure,Lamotrigin |
| 187 | Quetiapin,HCT,Trimipramin |
| 188 | Quetiapin,L-Thyroxin,ASS,Ramipril,Lorazepam,Amisulprid |
| 189 | Quetiapin,Lorazepam |
| 190 | Quetiapin,Mirtazapin |
| 191 | Quetiapin,Mirtazapin,Metoprolol mite |
| 192 | Ramipril,Acarbose,Glibenclamid,furosemide,ASS,Quetiapin |
| 193 | Ramipril,ASS,Metoprolol |
| 194 | Ramipril,Hydrochlorothiazid,Amlodipin,Mirtazapin |
| 195 | Ramipril,Mirtazapin |
| 196 | Reboxetin |
| 197 | Reboxetin,Mirtazapin,Quetiapin |
| 198 | Reboxetin,Propranolol,Lorazepam,Mirtazapin |
| 199 | Risperidon,Candesartan |
| 200 | Thephyllin |
| 201 | Topiramat,Aripiprazol |
| 202 | Trazodon,Propranolol |
| 203 | Trimipramin |
| 204 | Trimipramin |
| 205 | Trimipramin |
| 206 | Trimipramin,Pipamperon |
| 207 | Trimipramin,Reboxetin,Bisoprolol |
| 208 | Trospiumchlorid,Metoprolol,Ginkgo,Trimipramin,Risperidon |
| 209 | Valproat,Trimipramin |
| 210 | Valproat,Ziprasidon |
| 211 | Valproinsäure,Quetiapin,Buprenorphin,Trimipramin |
| 212 | Verapamil ret.,Zopiclon,Mirtazapin,Pantaprazol,Lorazepam,Latanoprost |
| 213 | Vit.B1,Magnesium,Kalium,Pantaprazol,Xipamide,Vitamin K |
| 214 | Vitamin B1,Magnesium,Mirtazapin |
| 215 | Vitamin B1,Magnesium,Venlafaxin,Ibuprofen,Folsäure,Mirtazapin |
| 216 | Vitamin D,Clopidogrel,Ramipril,Prednisolon,Verapamil,Alendronsäure |
| 217 | Zolpidem |
| 218 | Zopiclon,Chlorprothixen |
